# Express Your LOV: An Engineered Flavoprotein as a Reporter for Protein Expression and Purification

**DOI:** 10.1371/journal.pone.0052962

**Published:** 2012-12-27

**Authors:** Jayde A. Gawthorne, L. Evan Reddick, Snezhana N. Akpunarlieva, Katherine S. H. Beckham, John M. Christie, Neal M. Alto, Mads Gabrielsen, Andrew J. Roe

**Affiliations:** 1 Institute of Infection, Immunity and Inflammation, College of Medical, Veterinary and Life Sciences, University of Glasgow, Glasgow, United Kingdom; 2 Molecular Microbiology Department, University of Texas, Southwestern Medical Center, Dallas, Texas, United States of America; 3 Institute of Molecular, Cell and Systems Biology, College of Medical, Veterinary and Life Sciences, University of Glasgow, Glasgow, United Kingdom; 4 The Beatson Institute, Glasgow, United Kingdom; University of Pittsburgh, United States of America

## Abstract

In this work, we describe the utility of Light, Oxygen, or Voltage-sensing (LOV) flavoprotein domains from plant phototropins as a reporter for protein expression and function. Specifically, we used iLOV, an enhanced and more photostable variant of LOV. A pET-based plasmid for protein expression was constructed, encoding a C terminal iLOV-octahistidine (His8)-tag and a HRV 3C protease cleavage recognition site. Ten different proteins, with various sub-cellular locations, were cloned into the plasmid, creating iLOV-His8 tag fusions. To test protein expression and how iLOV could be used as a reporter, the proteins were expressed in three different cell lines, in four different culture media, at two different temperatures. To establish whether the presence of the iLOV tag could have an impact on the functionality, one of the proteins, EspG, was over-expressed and purified. EspG is an “effector” protein normally produced by enterohemorrhagic *E. coli* strains and “injected” into host cells via the T3SS. We tested functionality of EspG-iLOV fusion by performing functional studies of EspG in mammalian host cells. When EspG-iLOV was microinjected into the host cell, the Golgi apparatus was completely disrupted as had previously been observed for EspG.

## Introduction

The over-expression of recombinant proteins, whilst commonplace, is still an early bottleneck in biochemistry and structural biology. Although many proteins over-express easily in quantities suitable for both biochemical and structural studies, screening for optimal expression conditions is often required to maximise the output. The ability to quickly determine whether a protein is expressed under certain conditions without relying on more traditional methods of protein expression detection such as western blots of whole cell lysate, or partially purified proteins, is a great aid in high-throughput (HTP) expression studies. In this regard, fluorescent markers such as green fluorescent protein (GFP) and its derivatives have been utilised with great success [1], [2], [3], [4].

Fluorescent proteins offer great potential as effective and easy to use genetically encoded markers for protein expression and purification. The proliferation of publications utilising fluorescent proteins for this purpose has increased steadily (for review see [5]). A recent example is the novel infra-red protein, IFP, and its use in HTP expression screening of plant and fungal proteins in *Escherichia coli* [6]. Although the current range of fluorescent proteins cover almost the entire visible spectrum, GFP remains a popular choice due to its time to maturation, brightness and photostability [7]. However, GFP is not suitable for all applications due to its size (27 kDa) and structure (11 stranded β-barrel) and the reliance on an aerobic environment for fluorescence. Furthermore, GFP is not folded correctly when targeted to the periplasm of *E. coli* by a Sec-translocon signal peptide [8]. Therefore, there is considerable interest in developing alternative protein-based fluorophores that can be utilised under more complex HTP expression requirements, such as conditions of hypoxia.

One alternative reporter is the Light, Oxygen, or Voltage-sensing (LOV) flavoprotein domain from plant phototropins, which fluoresce in a similar part of the spectrum as GFP, upon blue light excitation [9]. The flavin chromophore of the LOV domain also provides it with a yellow colour that can be used conveniently to monitor protein purification [10]. Molecular evolution strategies have already been employed to improve the fluorescent properties of the LOV domain and enhance its viability as fluorescent reporter [11]–[12]. LOV-based fluorescent proteins are particularly beneficial, as their fluorescence is not dependent on the presence of oxygen [13] facilitating their utility under anaerobic environments. Moreover, no specialised microscope investments are necessary to monitor LOV fluorescence since its spectral properties overlap with those of GFP [11].

Here we present the use of a iLOV-tag [11] [10] as an alternative expression reporting fluorophore and describe the design of a novel construct, encoding a C-terminal iLOV-octahistidine (His_8_)-tag. Its small size (∼11 kDa) makes the iLOV-His_8_ tag ideal for monitoring protein over-expression and purification. The construct includes a HRV 3C protease cleavage recognition site located between the protein of interest and the iLOV-His_8_ tag allowing removal of the fluorescent domain.

To test the construct, 10 different proteins, with various sub-cellular locations, were encoded fused to the iLOV-His_8_ tag (Table 1). The proteins were expressed in three different cell lines, in four different culture media, at two different temperatures. The functionality of the cleavage site was confirmed, by over-expressing protein P3 (AdhE-D2-iLOV), followed by purification and cleaving using a His-tagged HRV 3C protease.

**Table 1:** Summary of the proteins used in this study to create the ten iLOV fusions.

Protein number	Protein name	Localisation	Functionality
P1	AdhE-iLOV	Cytosolic	Bifunctional Acetylaldehyde dehydrogenase/Alcohol dehydrogenase
P2	AdhED1-iLOV	Cytosolic	Acetylaldehyde dehydrogenase domain
P3	AdhED2-iLOV	Cytosolic	Alcohol dehydrogenase domain
P4	YhaO-iLOV	Inner membrane	Putative serine/threonine transporter
P5	YjdL-iLOV	Inner membrane	Peptide transporter
P6	EspG-iLOV	Secreted	Effector protein
P7	EspZ-iLOV	Secreted	Effector protein
P8	NleH1-iLOV	Secreted	Effector protein
P9	FklB-iLOV	Periplasmic	Peptidyl-prolyl cis-trans isomerase
P10	SurA-iLOV	Periplasmic	Peptidyl-prolyl cis-trans isomerase

To establish whether the presence of the iLOV-His_8_ tag could have an impact on the functionality, protein P6 (EspG-iLOV) was over-expressed and purified. We tested the EspG-iLOV fusion by repeating functional studies of EspG in mammalian host cells, as described by Seleyunin *et al.* that had shown disruption of the Golgi apparatus [14]–[15].

In this study we demonstrate the use of iLOV as a novel tool for the measurement of protein expression using HTP. We describe the use of the iLOV-tag as a reporter for protein expression and purification of proteins, confirm the functionality of our construct, and detail the work-flow for measuring expression using this system. Furthermore, we test the functionality using an effector protein fused to iLOV, and show the effector is active following microinjection into eukaryotic cells.

## Materials and Methods

### Cloning and construct

In order to allow complete control over the genetic sequence, DNA 2.0 (http://www.dna20.com) was used to synthesise the plasmid pET-iLOV-y*haO*, containing the *yhaO* gene from *Escherichia coli* TUV-930, a T7 promoter, a C3 protease site sequence and the region encoding the iLOV domain [11] [10]. The y*haO* gene is flanked by *Nco*I restriction sites allowing the gene to be excised and permitting in-frame cloning of other genes of interest into the linearised pET-iLOV vector. The use of gene synthesis also allowed codon optimisation of the iLOV gene to maximise potential expression in *E. coli*.

Targets were selected to represent as disperse a group of prokaryotic proteins as possible, represented by proteins normally located in the cytosol, the inner membrane and the periplasm. Additionally, proteins were included that are normally secreted through the T3SS (Table 1). However, all soluble proteins were cloned without their cognate signal sequence, to enhance cytosolic retention. Each gene was amplified with KOD hotstart polymerase (Promega) using the primers outlined in Table S1. The reactions were purified using a Qiagen PCR purification kit, digested with either *Nco*I or *Pci*I (New England Biolabs), and purified again. Purified PCR products and purified, linearised pET-iLOV vector were ligated using standard conditions. Ligations were transformed into Top10 chemically competent cells (Invitrogen) and the resulting plasmids were purified using a Qiagen mini prep kit. The new constructs were confirmed by PCR and sequencing. The plasmids generated throughout the study are detailed in Table S1.

### High-throughput testing for fluorescent protein expression

High-throughput expression was modified from previously published protocols [16]. Briefly, 10 proteins were over-expressed in 3 *E. coli* expression bacterial strains; C41(λDE3), BL21(λDE3) pLysS and Rosetta (λDE3) pLysS. Each bacterial strain was grown in 4 ml cultures of LB, TB, 2YT or M9 minimal media, in duplicated 48-well deep-well blocks (DWB). The bacterial strains and media were chosen as they are commonly used in modern laboratories [17]. After induction with 1 mM isopropyl-β-*D*-thiogalactopyranoside (IPTG), one DWB was moved to 25°C, whilst the other was maintained at 37°C. 100 µl samples were taken every hour, and the OD_600_ and the fluorescence intensity (emission and excitation wavelengths of 485 and 520 nm, respectively) was measured using a FLUOstar OPTIMA, BMG Labtech plate reader. After overnight incubation the cells were harvested by centrifugation at 3720 *g*. The cell pellets were suspended in 300 µl BugBuster (Novagen) and incubated at room temperature for 30 minutes to lyse. From the lysate, 20 µl samples were applied onto a nitrocellulose membrane and a dot-blot was performed, using His-probe HRP conjugate (Pierce) to detect the presence of a His_8_-tag.

### Data presentation

Heat-maps presenting data from the HTP experiments were created using Microsoft Excel (2003 or above, that supports a Visual basics application). The data was transferred from the fluorescence reader in an Excel spread-sheet format, and the rows and columns were organised as required. For convenience, the data was normalised to range from 0 to 1. The area of interest was highlighted, and a “surface contour” representation was selected; the scale of the z-axis can be adjusted. The heat-map was completed by running the macro (Appendix S1) in the VBA module within Excel.

### Scale up expression and purification

To further test the construct, and the use of iLOV as a marker for purification, two proteins, P3 (AdhED2-iLOV) and P6 (EspG-iLOV), were over-expressed in BL21(λ. DE3) pLysS cells, and grown in 2×500 ml LB cultures. Post-induction temperature was 25°C. The proteins were purified following established purification protocols [18]. The *espG* gene from EHEC O157:H7 was PCR cloned in-frame into pEGFP-C2 (Clontech) as previously described [15]. For bacterial expression 41 amino acid N-terminal deletions of EspG were used. The expression and purification of EspGΔ42 was performed as described elsewhere [15].

### Cleavage and separation of the His-iLOV tag

The iLOV-His_8_ tag was cleaved by adding Human Rhino Virus (HRV) 3C protease (1 mg protease per 50 mg P3-iLOV fusion) to the protein and the mix was dialysed against 20 mM Tris pH 7.6, 50 mM NaC, 5 mM DTT, at 4 °C overnight. The following day the solution was added to 1 ml Ni-NTA beads, pre-equilibrated in the dialysis buffer. The beads-protein mix was incubated for 1 hour, and the beads were collected by slow centrifugation at 1,000 *g* for 5 minutes. The colourless supernatant contained the cleaved protein of interest. Pre- and post-cleavage samples were applied onto a 10% Bis-Tris polyacrylamide gel for SDS-PAGE analysis.

### Testing the functionality of EspG when fused to iLOV

Normal Rat Kidney (NRK) cells were grown to ∼50% confluency in DMEM, 10% FBS, 1% Penicillin/Streptomycin on glass coverslips. Microinjection of EspG-iLOV (0.25 mg ml^−1^) or EspGΔ42 (0.25 mg ml^−1^) was carried out as previously described using Cascade Blue (1 mg ml^−1^) as tracking dye [19]. Cells were cultured for 45 min post-microinjection at 37°C, 5% CO_2_ and subsequently fixed with 3.7% formaldehyde and immunohistochemistry was performed following conventional protocols.

## Results

### Design of the pET-iLOV expression vector

**Figure 1:**
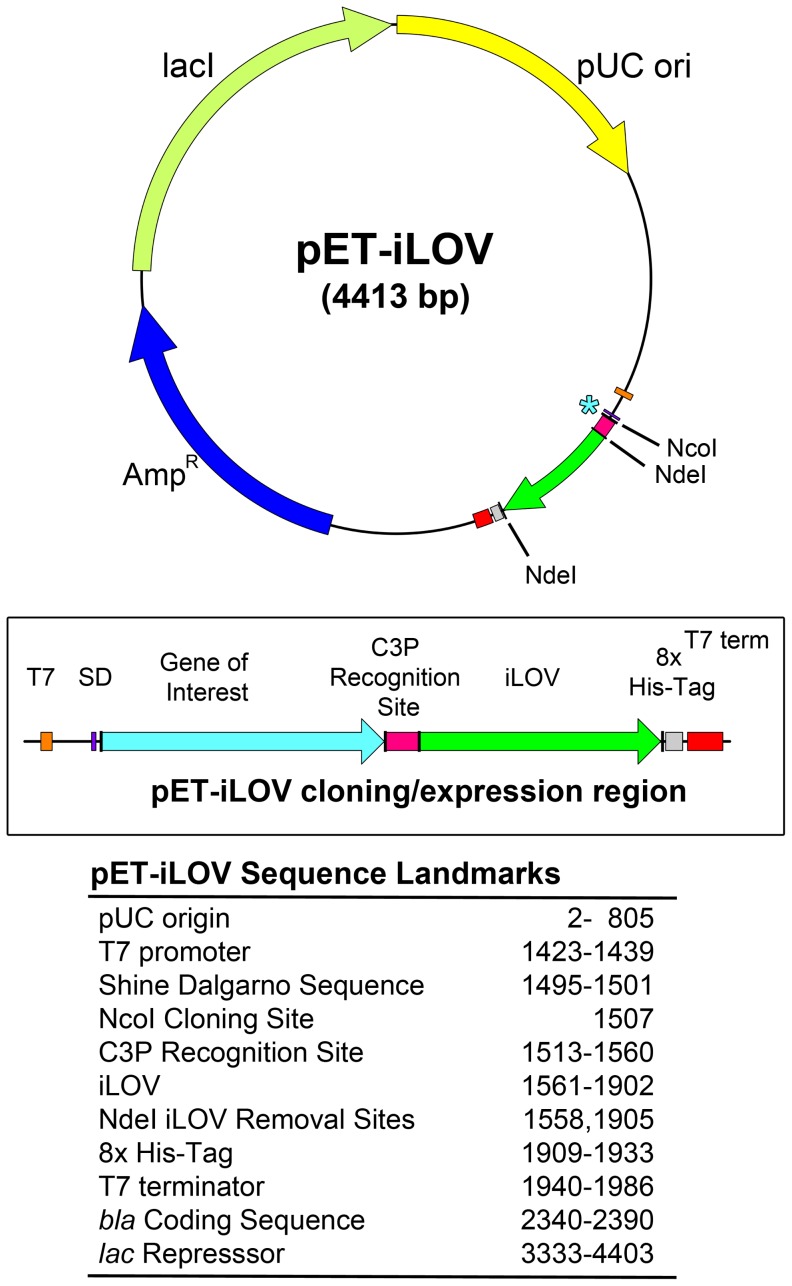
The pET-iLOV plasmid with key features highlighted including the iLOV domain (green) and key restriction sites for cloning genes of interest.

The pET-iLOV plasmid is based upon the established and widely utilized pET expression system, the “gold standard” for *in vivo* protein expression in *E. coli* (Figure 1). The gene of interest is cloned under the control of strong bacteriophage T7 transcription and translation signals and induction of expression via IPTG allows the protein of interest to comprise >50% total cell protein in a few hours. The promoter contains flanking palindromic lac operator binding sites that result in a 10-fold increase in *lac* repressor affinity *in vitro* relative to the natural promoter [20] and the Shine-Dalgarno sequence has been optimized for expression in *E. coli*.

### Expression of iLOV-tagged proteins

**Figure 2:**
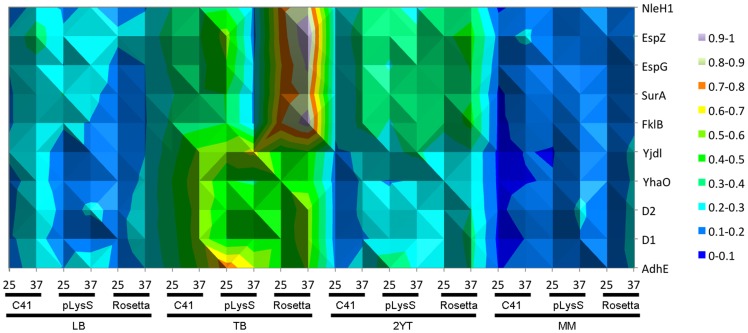
Heat-map of iLOV fluorescence for the 10 proteins analysed in this study. Each protein-iLOV fusion was cultured in four different media (LB, TB, 2YT and M9), three different cell lines (C41, pLysS, Rosetta) and at two different temperatures (25°C and 37°C). Peak fluorescence was determined during the growth curve. Fluorescence data were normalized from the absolute fluorescence values to a scale of 0 to 1. The area of interest was highlighted, and a “surface contour” representation was selected.

**Figure 3:**
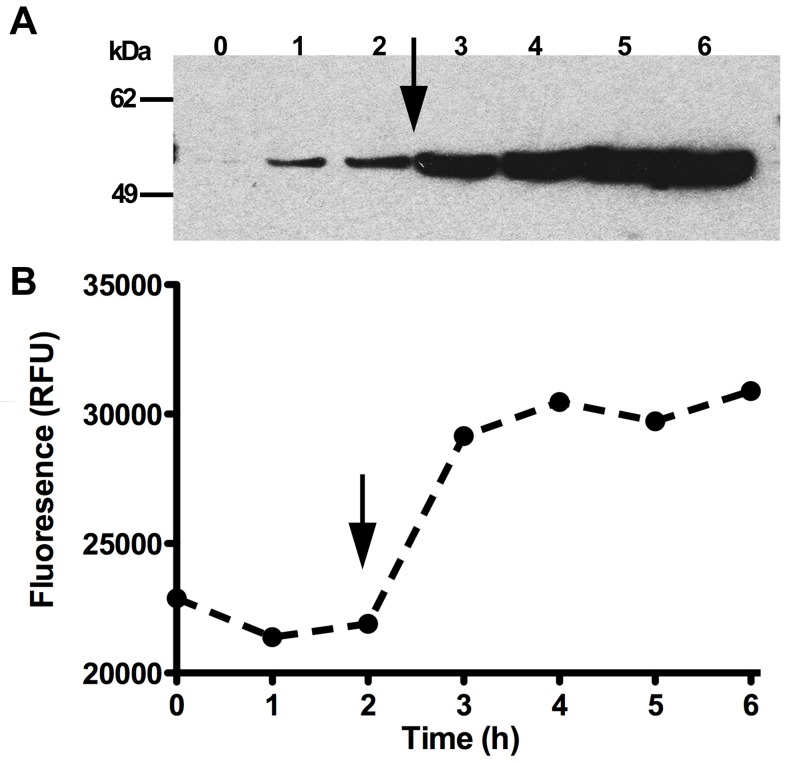
Western blot using an anti-iLOV antibody shows expression of the full-length AdhED2-iLOV-fusion protein (“P3”, A). Expression of AdhED2-iLOV-fusion protein (“P3”) over time corresponds with fluorescence, (B). AdhED2-iLOV was expressed in *E. coli* C41 cells and the level of fluorescence monitored. The arrow indicates addition of 1 mM IPTG to induce expression.

The expression levels of the iLOV-tagged proteins were monitored hourly and the final readings were taken on the next day, approximately 16 hours after induction. The OD_600_ readings showed that the cells grew in all media conditions, with the best growth in TB and 2YT (the richest media), and the least growth in M9 minimal media. Incubation temperature appears to have a protein and cell- line specific effect, as some conditions gave higher fluorescence readings at 25°C (e.g. P6 and P7 in C41 or pLysS cells), whereas proteins P1–P3 give higher fluorescence readings at 37°C. These results suggest that the temperature, cell line and media are parameters that should all be tested when approaching a new protein. To highlight successful expression of the iLOV-tagged proteins, the end-point fluorescence intensities are presented as a heat-map (Figure 2). The majority of the constructs produced fluorescing protein in at least one condition (8 of 10). To verify that fluorescence accurately reflected expression of the full-length fusion protein, we analysed P3 (AdhED2-iLOV) following induction by addition of IPTG. Western blotting using an anti-iLOV antibody revealed a band of 56 kDa corresponding to full-length P3 (Figure 3). No breakdown products were observed on the Western blot. Fluorescence of the bacteria expressing P3 was also monitored and assessed for the corresponding time points that were taken for Western analysis. This showed clear production of fluorescence following induction and a good correlation with the Western blot analysis (Figure 3). Of the two that failed to show any fluorescence, one is P8 (NleH1-iLOV), an effector protein that is translocated by pathogenic *E. coli* via the T3SS, and P4 (YhaO-iLOV), an inner membrane transporter, with the C-terminus located in the periplasm.

### Confirming the details of the construct – C3 Protease Cleavage

**Figure 4:**
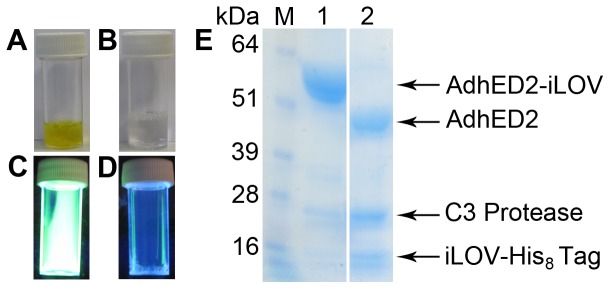
Purification of AdhE-D2-iLOV (P3) is easy to follow due to the distinctive yellow-green colour of the iLOV tagged protein, (A). Purified AdhE-D2 lacking the iLOV domain is colorless, (B). Purified P3 shows fluorescence under ultraviolet light, (C). Purified AdhE-D2 lacking the iLOV domain viewed under uv light (D). The iLOV domain can be readily cleaved from purified proteins using C3 protease, (E). Lane M: Markers, Lane 1: AdhE-D2-iLOV, indicated by arrow, Lane 2: AdhE-D2, iLOV and 3C protease indicated by arrows. The iLOV-His_8_ tag has been cleaved off, and the size of AdhE-D2 has been reduced by approximately 16 kDa.

To confirm that the construct performs as expected, P3 (AdhED2-iLOV) was expressed in a 500 ml culture. The protein over-expressed well, yielding approximately 3 mg, and eluted from the initial nickel column as a single green fluorescent fraction (Figure 4A–B). The correct fraction was further confirmed by UV illumination indicating the presence of AdhED2-iLOV, P3, (Figure 4C–D). The iLOV-tag was cleaved off by the addition of a His-tagged HRV C3 protease, and the mixture was analysed by SDS-PAGE. The molecular weight of the predominant band, AdhED2, was seen to drop by approximately 16 kDa. The liberated iLOV-His_8_-tag and His-tagged HRV C3 Protease are visualised at the bottom of the SDS-PAGE gel (Figure 4E). A second Ni-NTA step could be used to remove the iLOV-His_8_-tag and C3 Protease to further purify the protein.

### Confirming the negligible impact of the iLOV tag on the protein function

**Figure 5:**
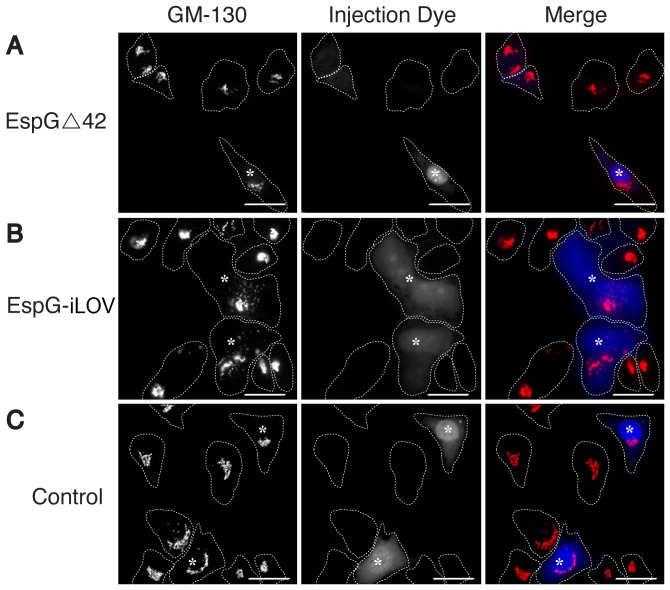
Microinjection of wild-type EspGΔ42 (A), EspG-iLOV (B), and buffer control (C), into cultured NRK cells. Visualized is the Golgi protein GM130, cascade blue injection dye, and merge as indicated in the vertical columns. Scale bar represents 50 µm.

EspG is an “effector” protein normally produced by enterohaemorrhagic *E. coli* strains and “injected” into host cells via the Type 3 Secretion System (T3SS). Effectors exert discrete activities on host cell processes with EspG being previously shown to target ADP-ribosylation factor (ARF) GTPases and p21-activated kinases (PAKs) in the host [14]. To determine whether the iLOV-His_8_ tag has an impact on the functionality of the protein, P6 (EspG-iLOV) was over-expressed, purified and microinjected into cultured mammalian cells. Previous studies used EspG Δ42 (deletion of 42 N-terminal residues), tagged with eGFP to show that EspG Δ42 efficiently disrupts the streaming of the eukaryotic Golgi complex, assessed by the observation of tubulovesicular Golgi remnants (Figure 5, Panel A–C). Similarly, the microinjection of full-length EspG-iLOV, resulted in rapid and robust disruption of the Golgi, with a phenotype nearly identical to that previously shown (Figure 5, Panel D–F). Injection of cascade blue dye alone did not alter the Golgi in any significant way (Figure 5, Panel G–I).

## Discussion

Here we report the use of the iLOV domain as an improved marker for protein expression and purification and its utility in the development of a robust HTP screen for a wide range of proteins expressed in *E. coli*. The use of a fluorescent tag, in particular GFP, as a marker of protein expression and purification is a common and very useful tool to produce protein for use in biophysical or biochemical studies [1], [2], [3], [4]. The main benefits of iLOV over GFP for protein expression and purification is that fluorescence is independent of the aerobic state, enabling correct folding of the fluorescent marker even in the absence of cellular oxygen [13], and the significantly smaller molecular weight, permitting functional studies to be undertaken using purified proteins with the iLOV-tag still attached. The utility of purified iLOV-tagged proteins for the determination of sub-cellular localization using appropriate microscopy techniques, is currently being investigated.

8 of the 10 fusion proteins fluoresce in our expression trials and these comprised a wide variety of proteins with different functions, structures and localisations. The high success rate of our tested constructs is indicative of the robust nature of the iLOV-tag as an alternative genetically encoded expression marker. The iLOV-tag, coupled to the His_8_-tag for ease of purification, has bright fluorescence enabling expression to be tracked over time using standard equipment, in this case a fluorometer. The peak fluorescence has been shown to correspond to the optimal expression of the construct being tested, providing a good framework for the HTP screen to be implemented for further proteins of interest. The vector used in this study contains a C-terminal iLOV-tag, however, the vector could easily incorporate an N-terminal tag if the protein of interest so required.

One of the two non-fluorescing fusions generated was NleH1-iLOV, an effector protein translocated by pathogenic *E. coli* via the T3SS and YhaO-iLOV, an inner membrane protein with a periplasmic C-terminus. Western analysis suggested that YhaO-iLOV is over-expressed in some of the conditions (data not shown). The lack of fluorescence in this construct is likely caused by the fact that YhaO-iLOV is inserted into the membrane via the Sec-translocon, which transports unfolded protein either into the membrane or through to the periplasm. Unfolding iLOV releases the flavin and upon refolding in the periplasmic space, the flavin is not accessible to be taken up by the iLOV, effectively preventing fluorescence. Alternatively, folded iLOV will not allow YhaO-iLOV to insert correctly into the membrane and may have been deposited in inclusion bodies in the cytoplasm. In the case of NleH1-iLOV, it is possible that the protein requires its cognate chaperone for proper folding or alternatively may only exhibit correct folding when “injected” into eukaryotic host cells. The remaining proteins that fluoresced encompassed an inner membrane protein, cytosolic proteins, periplasmic proteins and secreted effector proteins. It is worth bearing in mind that the periplasmic and effector proteins lack their leading sequence, and therefore remained in the cytosol. This is a common strategy in protein over-expression.

The success rate of our constructs suggests that iLOV will be a valuable tag for a wide variety of systems. The iLOV-His_8_ tag is fused to the gene of interest, separated by the sequence encoding a 3C Protease cleavage site, allowing for efficient removal of the genetically encoded tags. The fractions containing the iLOV-tag fused to the protein of interest appear bright yellow and can be tracked visually throughout the purification, allowing fast identification of protein containing fractions without the need for western analysis. The use of a pET based vector enabled all the reported positive features to be exploited for the HTP expression and purification system and provided a thoroughly tested framework to determine the utility and functionality of the new vector (for review see [21]). The ability to tightly control the expression of the desired protein is the main feature of the pET system we wished to exploit. This new vector will therefore be used to express a wide range of proteins, under a diverse range of conditions to ensure maximal output via the HTP screen proposed here.

Our results show that the iLOV-His_8_ tag can be ablated from the protein of interest, leaving the target protein cleaved and near wild type in sequence (an addition of PWGAGGLEVLFQ remains after the tag has been cleaved), to be separated from the iLOV-His_8_ tag and the protease by a secondary Ni-NTA column. To determine any potential impact the iLOV-His_8_ tag could have on protein functionality, EspG-iLOV and EspGΔ42 were over-expressed and microinjected into NRK cells. Heterologously expressed EspG Δ42 fused with either GFP or GST has been shown to be capable of Golgi disruption. This observed organelle disruption is caused by the targeting of EspG to the GTP-bound form of the ARF1 GTPase and the subsequent prevention of the natural nucleotide cycle [15]. Our results confirm these findings, and highlights that the addition of the iLOV domain to EspG does not affect the activity of EspG *in vivo*. This result indicates that purified iLOV-tagged fusion proteins from the pET-iLOV vector described here can be easily purified, delivered to target cells and can be expected to perform in a manner similar to the wild-type protein. Whilst the fluorescence of EspG-iLOV was not at a sufficient level to be detected after microinjection, neither was the EspG-eGFP variant used in the study. However, as recent papers suggest [12], the development of iLOV as a new fluorophore is just beginning and newer, brighter, more photostable variants could easily be incorporated into our pET-iLOV vector as they are developed and thoroughly tested.

The iLOV-tag utilised here displays amino acid sequence homology to the newly reported miniSOG, a light and electron microscopy fusable marker [22]. Whether iLOV will also produce singlet oxygen, similar to miniSOG, is yet to be determined. However, as many of the key active sites overlap with iLOV the potential for visualisation with both light and electron microscopy (EM) exists. Therefore the possibility for correlative EM (CLEM) of proteins linked to the iLOV-tag either expressed *in vivo* or purified and microinjected makes the HTP screen an attractive method of screening expression of proteins of interest for this purpose. The potential applications for iLOV-tagged purified proteins in visual experimentation is wide, ranging from dissecting complex host-pathogen interactions to further understanding specific eukaryotic cell functions applicable to important disease states.

## Supporting Information

Table S1 **List of oligonucleotides and plasmids in the study.** Enzymatic restriction sites are underlined.
(DOCX)

Appendix S1 **Macro for generation of the heatmaps using Visual Basic for Applications (VBA) in Excel.**
(DOCX)
