# Supplementary material for: Express Your LOV: An Engineered Flavoprotein as a Reporter for Protein Expression and Purification
Source: PLoS One. 2012 Dec 27;7(12):e52962. doi: 10.1371/journal.pone.0052962 (PMC3531456; doi:10.1371/journal.pone.0052962)
Supplement: Table S1 — List of oligonucleotides and plasmids in the study. Enzymatic restriction sites are underlined. (DOCX) [file pone.0052962.s001.docx]

Table S1.

| **Primer Name** | **Application** | **Sequence 5’ -> 3’** |
| --- | --- | --- |
| p77Rev | Verification of pET-iLOV fusions | GTTAGTGATGGTGATGATGG |
| AdhED1_5’ | Creation of pET-AdhED1-iLOV (P2) and pET-AdhE-iLOV (P1) | ACCATGGACATGGCGGTTACCAATGTTG |
| AdhED1_3’ | Creation of pET-AdhED1-iLOV (P2) | ACCATGGCCGACGAAGATAGATGCTT |
| AdhED2_5’ | Creation of pET-D2-iLOV (P3) | accatggacatgctgtggcataagctgcc |
| AdhED2_3’ | Creation of pET-AdhED2-iLOV (P3) and pET-AdhE-iLOV (P1) | ACCATGGCGCGGATTTCTTC |
| YjdL_5’ | Creation of pET-YjdL-iLOV (P5) | Ccatggaaaaaacaccctcacagc |
| YjdL_3’ | Creation of pET-YjdL-iLOV (P5) | CCATGGATCGTTGCTCTCCTGTACCA |
| EspG_5’ | Creation of pET-EspG-iLOV (P6) | GGCCATGGTACTTGTTGCCAAATTGTTCATTAC |
| EspG_3’ | Creation of pET-EspG-iLOV (P6) | GGCCATGGAGTGTTTTGTAAGTACGTTTCAGATGC |
| EspZ_5’ | Creation of pET-EspZ-iLOV (P7) | GGCCATGGAAGCAGCAAATTTAAGTCC |
| EspZ_3’ | Creation of pET-EspZ-iLOV (P7) | GGCCATGGGGCATATTTCATCGCTAATGCACC |
| NleHI_5’ | Creation of pET-NleHI-iLOV (P8) | GGACATGTTATCGCCATATTCTGTAAATTTGG |
| NleHI_3’ | Creation of pET-NleHI-iLOV (P8) | GGCCATGGAATTTTACTTAATACCACACTAA |
| FklB_5’ | Creation of pET-FklB-iLOV (P9) | AACCATGGTTAGTTGCTCAGGATTTTAA |
| FklB_3’ | Creation of pET-FklB-iLOV (P9) | CCCCATGGATGAAGAACTGGAAACGCT |
| SurA_5’ | Creation of pET-SurA-iLOV (P10) | TGCCATGGTTAGAGGATTTCCAGGAGTT |
| SurA_3’ | Creation of pET-SurA-iLOV (P10) | GACCATGGATGACCACCCCAATTTTGA |
