# Supplementary material for: Express Your LOV: An Engineered Flavoprotein as a Reporter for Protein Expression and Purification
Source: PLoS One. 2012 Dec 27;7(12):e52962. doi: 10.1371/journal.pone.0052962 (PMC3531456; doi:10.1371/journal.pone.0052962)
Supplement: Appendix S1 — Macro for generation of the heatmaps using Visual Basic for Applications (VBA) in Excel. (DOCX) [file pone.0052962.s002.docx]

Sub RemoveLinesandSetColors()

Dim LE As Integer

Dim red As Integer

Dim green As Integer

Dim blue As Integer

Dim a As Integer

Dim x As Integer

Dim y As Integer

Dim z As Integer

Dim q As Integer

red = 0

green = 0

blue = 255

LE = 1

If ActiveChart Is Nothing Then Exit Sub

If MsgBox("Remove lines from surface chart and set colors?", vbYesNo) = vbYes Then Application.ScreenUpdating = False ActiveChart.HasLegend = True a = ActiveChart.Legend.LegendEntries.Count

x = a / 4

y = Int(255 / x)

For z = 1 To 4

For q = 1 To

ActiveChart.Legend.LegendEntries(LE).LegendKey.Border.LineStyle = xlNone

ActiveChart.Legend.LegendEntries(LE).LegendKey.Interior.Color = RGB(red, green, blue)

If z = 1 Then green = green + y

If z = 2 Then blue = blue - y

If z = 3 Then red = red + y

If z = 4 Then green = green - y

LE = LE + 1 Next q

q = 1 Next z

End If

End Sub
